# Twenty years of inhaled insulin: promise, setbacks, and future directions

**DOI:** 10.17179/2025-8260

**Published:** 2025-05-28

**Authors:** Meriem Gaddas, Imen Ben Saida, Helmi Ben Saad

**Affiliations:** 1 University of Sousse, Faculty of Medicine 'Ibn el Jazzar' of Sousse, Farhat HACHED University Hospital, Research Laboratory LR12SP09 'Heart Failure' Sousse, Tunisia; 2 Department of Physiology and Functional Explorations, Farhat HACHED University Hospital, Sousse, Tunisia; 3 Intensive Care Department, Farhat Hached University Hospital, Sousse, Tunisia

## ⁯

Insulin-dependent diabetes mellitus (DM) is a severe chronic condition requiring continuous medical management to prevent life-threatening complications (ADAPPC, 2025[1]). The cornerstone of treatment is insulin therapy, which is essential to maintain glycemic control in patients with this condition (Mishra et al., 2021[19]). In daily practice, insulin is primarily administered via subcutaneous injections, a method that, while effective (WHO, 2021[25]), presents several inconveniences, including lipodystrophy, pain, and allergic reactions, which can affect insulin absorption and efficacy (Maikawa et al., 2021[16]). Consequently, alternative delivery methods, including inhaled insulin, have been explored to improve patient outcomes and adherence to treatment (Cheng et al., 2021[8]). Research on inhaled insulin are few, and on February 5, 2025, a request based on (inhaled insulin [Title]) found 262 papers indexed in PubMed. These papers were published between 1997 and 2024 (ie; annual rate of 2.2 papers), and the peaks of papers were in 2005 and 2006 (n=42 each). This editorial reviewed the 20-year evolution of inhaled insulin, highlighting its benefits, limitations, and challenges. It also explored its future in the context of innovations in DM management.

The first inhaled insulin (Exubera), released in 2006, was approved by the Food and Drug Administration and launched with great fanfare (Bellary and Barnett, 2006[4]). It was presented as an alternative to cumbersome subcutaneous injections, with comparable efficacy and absorption (Perera et al., 2002[21]), and promising great comfort and adherence for patients (Cunningham and Tanner, 2020[9], Rave et al., 2005[24]). The inhaled route of insulin administration has the advantage of a large, well-irrigated exchange surface (ie; the alveolar-capillary surface area, estimated at 75-100 m^2^ (Borghardt et al., 2018[6]), enabling efficient absorption and a reduction in systemic side-effects (Borghardt et al., 2018[6]). The low metabolic activity of the lungs provides protection against the peptide degradation (Borghardt et al., 2018[6]), which is the main obstacle to the development of oral insulin (Wong et al., 2016[26]). In addition, respiratory administration avoids the hepatic degradation of insulin, which can be as high as 80%, during the 'first pass' (Meier et al., 2005[18]). However, a year after it was launched, Exubera had not met with the commercial success expected, leading to its withdrawal and a drop in interest in development projects in this area. This setback was attributed to its high cost, the complexity of its administration, and the appearance of contraindications in certain populations (Rashid et al., 2015[23]).

The experiment was repeated in 2014, with Afrezza (Technospher insulin), which was presented as a major innovation (Goldberg and Wong, 2015[11]). Disappointment with this compound was swift, given the successive complaints about its bioavailability and, above all, its therapeutic safety (Goldberg and Wong, 2015[11]). On the one hand, despite its faster onset (ie; 7-15 minutes), Afrezza had a short duration of action (ie; 2-6 hours) (Grant et al., 2022[12]), and exhibited inter-individual variability, necessitating frequent adjustments and even the combined use of other forms of insulin (Goldberg and Wong, 2015[11]). On the other hand, Afrezza had pulmonary irritation effects, requiring spirometry to be performed before initiating treatment (Mudaliar and Henry, 2007[20]). Almost two years after its launch, serious reservations had been expressed about the potential deleterious effects of Afrezza on lung function, involving a decline in spirometric data, with suspicions of an increased risk of lung cancer, particularly in smokers (McGill et al., 2020[17]). This fear may have been legitimate insofar as it has been established that insulin medication is significantly associated with the risk of cancer (Chen et al., 2023[7], Zhong and Mao, 2022[28]). On this point, it should be emphasized that the association between inhaled insulin and lung cancer has never been demonstrated, and these fears are based on the like-growth factor property of insulin (Rashid et al., 2015[23]). In addition, this hypothetical association stems from observations in a clinical trial of Exubera, where two patients with a history of heavy smoking developed cancer (McGill et al., 2020[17]). Several studies dating from after 2020 had demonstrated the absence of any significant long-term carcinological risk associated with the use of inhaled insulin (Afrezza in particular) (Greene et al., 2021[13]). These results were demonstrated in animals (Greene et al., 2021[13]), and approved in humans by a clinical trial involving 5500 patients, in which Afrezza's adverse pulmonary effects were comparable to those attributed to other treatments, with the exception of irritant cough (McGill et al., 2020[17]). Similarly, the decline in respiratory function would be of a reversible functional nature, with no objective organic support by radiology (McGill et al., 2020[17]). However, although the most-updated data would suggest an acceptable level of safety, it remains preferable to retain a warning notice for patients with pre-existing chronic respiratory conditions (Afreza, 2023[2]) (Box 1).


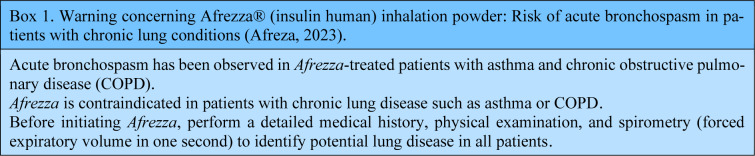


Compared with injectable insulin, inhaled insulin has a lower overall bioavailability due to losses in the airways, making it necessary to increase doses (Bellary and Barnett, 2006[4]). The bioavailability of inhaled insulin may be influenced by chronic respiratory conditions such as asthma and chronic obstructive pulmonary disease (COPD) (Mudaliar and Henry, 2007[20]). In fact, absorption is thought to be 30-40% lower in asthmatics (Mudaliar and Henry, 2007[20]), which means that a bronchodilator needs to be administered before inhaled insulin to optimize absorption, and doses need to be increased (Petersen et al., 2010[22]). In COPD patients, the absorption of inhaled insulin is described as variable (Mudaliar and Henry, 2007[20]). Smoking has a significant influence on the absorption of inhaled insulin, since smokers have faster and greater absorption (Becker et al., 2006[3]). This latter effect is reversible after smoking cessation (Becker et al., 2006[3]). This explains why inhaled insulin was not recommended for diabetic patients who continue to smoke (Becker et al., 2006[3]). A 2022 systematic review and meta-analysis of 13 low-bias randomized clinical trials comparing inhaled insulin with conventional insulin found that inhaled insulin is as effective as subcutaneous insulin in patients with type 1 DM (Khan et al., 2022[15]). Inhaled insulin was also associated with less weight gain and fewer hypoglycemic episodes, with similar effects on blood glucose levels and no increased risk of adverse events (Khan et al., 2022[15]). These findings suggest that inhaled insulin may be a suitable alternative for patients concerned about injection compliance or weight gain.

Recent trials suggest that inhaled insulin holds promise as an alternative for DM management (Hirsch et al., 2024[14]). Inhaled insulin currently appears to be most effective for achieving optimal post-meal blood sugar control when used in conjunction with basal insulin injections (Hirsch et al., 2024[14]). The Phase 4 INHALE-3 trial (MannKind) demonstrated that Afrezza plus basal insulin injections resulted in better post-meal glucose excursions compared to usual care (Hirsch et al., 2024[14]). Furthermore, preliminary data from the Phase 3, open-label, randomized INHALE-1 study evaluating Afrezza in combination with basal insulin in pediatric DM are also promising (Hirsch et al., 2024[14]). Ongoing long-term safety trials conducted by MannKind will further assess the safety and tolerability of Afrezza in adults with type 1 or type 2 DM, providing additional data on its long-term effects, particularly on the lungs (Hirsch et al., 2024[14]).

There are numerous advances on the market in the formulation of recombinant insulins, which aim to optimize the management of DM while also optimizing patients' quality of life (Bolli et al., 2022[5], Maikawa et al., 2021[16]). These formulations are potential “competitors” to inhaled insulin, such as rapid- and long-acting human insulin analogues, which closely mimic the physiological secretory profile of insulin (Bolli et al., 2022[5]), weekly insulin (AFRE) (Bolli et al., 2022[5]), and injection pens, which are more convenient and increasingly painless (Bolli et al., 2022[5]). The challenge of new inhaled insulin formulations is to allow a macromolecule such as insulin to pass through a pulmonary epithelial barrier specifically designed to stop the intrusion of exogenous agents (Rashid et al., 2015[23]). Among these innovative formulations are those based on advances in nanotechnology through the use of nanoparticles (Zhang et al., 2021[27]). The latter are agents for encapsulating insulin, aimed at improving its safety during transepithelial transport and absorption, which would guarantee greater stability (Zhang et al., 2021[27]). Some of these formulations would also allow control of the release time of the encapsulated insulin (rapid or long-lasting effect) (Zhang et al., 2021[27]). Although promising, inhaled nanopeptide insulins are still not approved for clinical use (Zhang et al., 2021[27]). There are certain limitations to their commercialization in terms of cost and long-term safety (eg; challenges relating to the stability of formulations and the disposal of materials used), since these are molecules in the experimental phase, under evaluation (Zhang et al., 2021[27]).

In conclusion, despite all the efforts made over the last twenty years (ie; 2004-2024) to develop inhaled insulins, these efforts have not been crowned with success, as the bioavailability of these preparations remains low and unpredictable compared with injectable insulin. This lack of success is likely further compounded by poor publicity and marketing since market release, particularly the historical failure of Exubera, which has contributed to hesitancy among patients and prescribers.


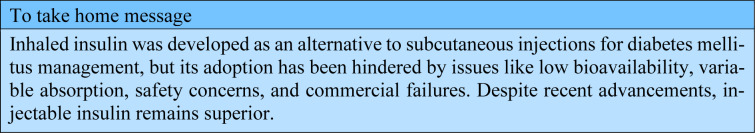


## Declaration

The authors wish to disclose that an artificial intelligence tool (i.e., ChatGPT 3.5 ephemeral) was utilized to enhance the manuscript's wording, readability, and language quality. The tool was used only for language refinement and not for generating text (Dergaa and Ben Saad, 2023[10]).

## References

[1] ADAPPC (2025). American diabetes association professional practice committee. 1. Improving care and promoting health in populations: Standards of care in diabetes-2025. Diabetes Care.

[2] Afreza (2023). Afreza's medication leaflet. https://afrezza.com/wp-content/uploads/2023/02/Full-Prescribing-Information-Feb-2023-1.pdf.

[3] Becker RH, Sha S, Frick AD, Fountaine RJ (2006). The effect of smoking cessation and subsequent resumption on absorption of inhaled insulin. Diabetes Care.

[4] Bellary S, Barnett AH (2006). Review: Inhaled insulin: overcoming barriers to insulin therapy?. Br J Diabetes Vasc Dis.

[5] Bolli GB, Cheng AYY, Owens DR (2022). Insulin: evolution of insulin formulations and their application in clinical practice over 100 years. Acta Diabetol.

[6] Borghardt JM, Kloft C, Sharma A (2018). Inhaled therapy in respiratory disease: The complex interplay of pulmonary kinetic processes. Can Respir J.

[7] Chen Y, Mushashi F, Son S, Bhatti P, Dummer T, Murphy RA (2023). Diabetes medications and cancer risk associations: a systematic review and meta-analysis of evidence over the past 10 years. Sci Rep.

[8] Cheng R, Taleb N, Stainforth-Dubois M, Rabasa-Lhoret R (2021). The promising future of insulin therapy in diabetes mellitus. Am J Physiol Endocrinol Metab.

[9] Cunningham SM, Tanner DA (2020). A review: The prospect of inhaled insulin therapy via vibrating mesh technology to treat diabetes. Int J Environ Res Public Health.

[10] Dergaa I, Ben Saad H (2023). Artificial intelligence and promoting open access in academic publishing. Tunis Med.

[11] Goldberg T, Wong E (2015). Afrezza (Insulin Human) inhalation powder: A new inhaled insulin for the management of type-1 or type-2 diabetes mellitus. P T.

[12] Grant M, Heise T, Baughman R (2022). Comparison of pharmacokinetics and pharmacodynamics of inhaled technosphere insulin and subcutaneous insulin lispro in the treatment of type 1 diabetes mellitus. Clin Pharmacokinet.

[13] Greene SF, Nikula KJ, Poulin D, McInally K, Reynolds JA (2021). Long-term nonclinical pulmonary safety assessment of Afrezza, a novel insulin inhalation powder. Toxicol Pathol.

[14] Hirsch IB, Beck RW, Marak MC, Calhoun P, Mottalib A, Salhin A (2024). A Randomized comparison of postprandial glucose excursion using inhaled insulin versus rapid-acting analog insulin in adults with type 1 diabetes using multiple daily injections of insulin or automated insulin delivery. Diabetes Care.

[15] Khan AB, Ahmad A, Ahmad S, Gul M, Iqbal F, Ullah H (2022). Comparative analysis of inhaled insulin with other types in type 1 diabetes mellitus: A systematic review and meta-analysis. Cureus.

[16] Maikawa CL, d'Aquino AI, Lal RA, Buckingham BA, Appel EA (2021). Engineering biopharmaceutical formulations to improve diabetes management. Sci Transl Med.

[17] McGill JB, Peters A, Buse JB, Steiner S, Tran T, Pompilio FM (2020). Comprehensive pulmonary safety review of inhaled technosphere((R)) insulin in patients with diabetes mellitus. Clin Drug Investig.

[18] Meier JJ, Veldhuis JD, Butler PC (2005). Pulsatile insulin secretion dictates systemic insulin delivery by regulating hepatic insulin extraction in humans. Diabetes.

[19] Mishra V, Nayak P, Sharma M, Albutti A, Alwashmi ASS, Aljasir MA (2021). Emerging treatment strategies for diabetes mellitus and associated complications: An update. Pharmaceutics.

[20] Mudaliar S, Henry RR (2007). Inhaled insulin in patients with asthma and chronic obstructive pulmonary disease. Diabetes Technol Ther.

[21] Perera AD, Kapitza C, Nosek L, Fishman RS, Shapiro DA, Heise T (2002). Absorption and metabolic effect of inhaled insulin: intrapatient variability after inhalation via the Aerodose insulin inhaler in patients with type 2 diabetes. Diabetes Care.

[22] Petersen AH, Korsatko S, Kohler G, Wutte A, Olschewski H, Sparre T (2010). The effect of terbutaline on the absorption of pulmonary administered insulin in subjects with asthma. Br J Clin Pharmacol.

[23] Rashid J, Absar S, Nahar K, Gupta N, Ahsan F (2015). Newer devices and improved formulations of inhaled insulin. Expert Opin Drug Deliv.

[24] Rave K, Bott S, Heinemann L, Sha S, Becker RH, Willavize SA (2005). Time-action profile of inhaled insulin in comparison with subcutaneously injected insulin lispro and regular human insulin. Diabetes Care.

[25] WHO (2022). World Health Organisation guideline on self-care interventions for health and well-being, 2022 revision. https://www.who.int/publications/i/item/9789240052192.

[26] Wong CY, Martinez J, Dass CR (2016). Oral delivery of insulin for treatment of diabetes: status quo, challenges and opportunities. J Pharm Pharmacol.

[27] Zhang T, Tang JZ, Fei X, Li Y, Song Y, Qian Z (2021). Can nanoparticles and nano‒protein interactions bring a bright future for insulin delivery?. Acta Pharm Sin B.

[28] Zhong W, Mao Y (2022). Daily insulin dose and cancer risk among patients with type 1 diabetes. JAMA Oncol.

